# Detection of Malignant Skin Lesions Based on Decision Fusion of Ensembles of Neural Networks

**DOI:** 10.3390/cancers15204946

**Published:** 2023-10-11

**Authors:** Loretta Ichim, Razvan-Ionut Mitrica, Madalina-Oana Serghei, Dan Popescu

**Affiliations:** 1 Faculty of Automatic Control and Computers, University Politehnica of Bucharest, 060042 Bucharest, Romania; loretta.ichim@upb.ro (L.I.); razvanionutm@yahoo.ro (R.-I.M.); madalina.serghei27@gmail.com (M.-O.S.); 2 “Ștefan S. Nicolau” Institute of Virology, 030304 Bucharest, Romania

**Keywords:** skin lesion detection, image processing, neural networks, decision fusion, neural networks ensemble

## Abstract

**Simple Summary:**

Due to various causes, such as the thinning of the ozone layer, climate change, or the fashion for artificial tanning, the incidence of skin cancer has increased recently. Early detection of cancerous skin lesions is the only chance to prevent dangerous developments. This paper proposes two models of support systems for the detection of some skin lesions including dangerous melanoma, based on two different methods of assembling neural networks and making global decisions by fusing individual decisions. The system based on the fusion of some intermediate binary classification subsystems is a new solution and offers an accuracy of 91.04%, better than that offered by the classic system based on the vote with multiple weights of the constituent networks. In addition, it is found that the individual performance indicators depend on the type of skin lesion. For example, the F1 score varied from 81.36% to 94.17%.

**Abstract:**

Today, skin cancer, and especially melanoma, is an increasing and dangerous health disease. The high mortality rate of some types of skin cancers needs to be detected in the early stages and treated urgently. The use of neural network ensembles for the detection of objects of interest in images has gained more and more interest due to the increased performance of the results. In this sense, this paper proposes two ensembles of neural networks, based on the fusion of the decisions of the component neural networks for the detection of four skin lesions (basal cancer cell, melanoma, benign keratosis, and melanocytic nevi). The first system is based on separate learning of three neural networks (MobileNet V2, DenseNet 169, and EfficientNet B2), with multiple weights for the four classes of lesions and weighted overall prediction. The second system is made up of six binary models (one for each pair of classes) for each network; the fusion and prediction are conducted by weighted summation per class and per model. In total, 18 such binary models will be considered. The 91.04% global accuracy of this set of binary models is superior to the first system (89.62%). Separately, only for the binary classifications within the system was the individual accuracy better. The individual F1 score for each class and the global system varied from 81.36% to 94.17%. Finally, a critical comparison is made with similar works from the literature.

## 1. Introduction

Skin cancer represents one of the forms of cancer with the highest prevalence and mortality rate, according to statistics from the American Cancer Society. The skin is the largest organ in the human body. Approximately one-third of the total number of cancer cases is represented by skin cancer. This type of cancer is in the epidermis and the main factor that causes it is long exposure to ultraviolet (UV) radiation, which represents the cause for approximately 90% of skin cancer cases [1]. The most lethal type of cancer present in humans is melanoma. Fortunately, there is a possibility for the patient to be cured if the disease is identified in its early stages, with the probability of survival being significant in these conditions. The occurrence of melanoma is favored by certain risk factors, such as repeated skin burns due to the sun, weakened immune system, hereditary factors, and the use of tanning beds. The main external factor that causes the appearance of melanoma on people’s skin is exposure to UV radiation emitted by the sun. Cancer occurs because of skin cells whose DNA (deoxyribonucleic acid) is affected by radiation, and which cannot heal. In the case of melanoma, cancer cells appear due to moles that lead to inflammation around the epidermis, which in turn leads to an increase in temperature in this area [1].

Basal cancer cells (basal cancer cell—bcc) are the least aggressive type of non-melanoma cancer. This form of cancer appears, mainly, in the areas most exposed to the sun—face, neck, arms, and legs—and can spread to other areas of the body and can grow in nerves and bones. Fortunately, although it is a cancer that occurs in over 2 million people annually, it can be effectively cured by simple methods.

Melanoma (mel) arises from melanocytes and represents the type of skin cancer with the fastest growth and, at the same time, the highest mortality rate. It can still be cured if identified in the early stages. It is usually treated by chemotherapy and radiation-based therapy or by prompt extirpation in the early stages. According to [2], the 5-year survival rate for melanoma cases is 99% in the case of its rapid detection. This percentage drops to 68% once the disease reaches the lymph nodes. In cases of late detection, once the disease has metastasized to other organs, the survival rate drops to 30%.

In this paper, two other types of lesions will be used as support for the identification of skin lesions, namely benign keratosis (bkl) and melanocytic nevi (nv), mainly due to their larger number of presences in the dataset used.

Melanocytic nevi are benign neoplasms that appear in many different forms from a dermatoscopic point of view, and benign keratoses represent a generic class that includes three other subclasses of lesions that, although they look different, are biologically similar. One of these subclasses is more difficult to identify due to certain morphological features that resemble those of melanoma.

Detecting skin cancer in the early stages is very important for increasing the probability of survival. The most accurate way of diagnosis is a biopsy. This is an invasive method that involves higher costs but also brings risks to the development of various infectious diseases. Another method that represents the standard today is the visual analysis performed by a specialist [3]. This method is expensive in terms of time and can lead to an erroneous diagnosis. The accuracy obtained in the identification of melanoma by this method is approximately 80%, and the results have low repeatability from one doctor to another.

Over time, numerous methods have been devised to diagnose skin lesions to identify melanoma. Many of these have the advantage of being non-invasive. In [1,4], a large part of these methods is presented in detail along with performance metrics. Spectroscopy based on electrical impedance involves measuring the conductivity of the lesion, which is greatly influenced by water content. The conductivity of the tissue depends on histological characteristics that change when the cells undergo malignant transformations. This method is only useful for identifying lesions in the early stages and through multiple measurements along the evolution. Fluorescence-based spectroscopy can differentiate cancerous from non-cancerous cells. The radiation used for excitation has a wavelength of 300 nm. Multispectral imaging is another method that can be used for detection in the early stages. This is based on the creation of 10 images with different spectra that can penetrate up to 2.5 mm deep below the surface of the skin. This method is used to decide whether a lesion requires a biopsy. Thermography is another method of diagnosing skin cancer. A higher measured temperature represents the presence of skin cancer.

The above methods presented different disadvantages regarding the performance indicators. As a result of technological developments in the last decade, a new class of skin cancer identification methods appeared and was successfully developed. Both for the purpose of classification and segmentation of skin lesions, the new trend is the use of artificial intelligence using neural networks or deep learning techniques.

Artificial intelligence (AI) tries to imitate the learning processes of the human brain. Initially, machine learning (ML) algorithms were developed to identify melanoma by extracting features and learning, in a supervised manner (most of the time), to classify the features into certain categories [3]. Several limitations (errors in certain situations and long training time) led to the development of algorithms based on neural networks (NNs). A neural network is made up of several neurons connected in the form of a network. Each such connection has an assigned weight, which will be optimized during the training phase. The final mathematical model is made up of all these weights that are modified based on the system error, which is calculated as the difference between the predicted value and the real value. Among the NN advantages is the lack of need for a large amount of information for the training stage, parallel processing capacity, and distributed memory.

One of the biggest difficulties in processing images with skin lesions (SLs) is the presence of noise and artifacts, most often in the form of hair. In [5], a conventional encoder–decoder architecture was proposed for hair removal from dermatoscopic images. The model made up of 12 layers was trained using pairs consisting of a dermatoscopic image and another image with simulated hair. By defining a loss function customized to the problem, the experimental results were at the state-of-the-art level. Apart from the classic noise removal methods, such as filters based on the mean or median value for image denoising, several methods based on NNs were recently proposed [5]. The same situation occurs in the case of hair removal from dermatoscopic images. In addition to classic methods, such as DullRazor, methods based on neural networks, such as Generative Adversarial Networks, U-Net, etc., are successfully used today. The noise removal methods as well as the hair (artifacts) removal methods from dermatoscopic images have a high degree of generalization and can be applied to other skin lesions (like acral lesions, lesions with an absence of surrounding normal skin, and in which there is an absence of pigmentation) as well.

The objective of this work is to train some neural networks for the purpose of identifying melanoma-type lesions and classifying several classes of lesions. To improve the classification performance, we chose an approach based on assembly techniques by voting. In more detail, this process involves the training of several neural networks and the assembly in different ways of the individual values predicted by these networks. Intervention in the structure of neural networks or the development of a new neural network was avoided. Thus, the purpose of this paper is to explore the capacity of assembly methods by voting for classification objectives, namely, to classify skin lesions considering four classes: bcc, mel, bkl, and nv. In this sense, two methods of assembling three neural networks (MobileNet [6], DenseNet [7], and EfficientNet [8]) are presented, to obtain higher accuracy.

## 2. Related Works

During the last 10 years, methods based on deep learning neural networks have become very popular in the classification and segmentation of SLs. Different systems and NN improvements have been proposed over the years to increase identification and classification performance. Three main tasks of NN applications were investigated: detection, classification, and segmentation. Considering that melanoma is the most dangerous SL, a comprehensive review of new trends of melanoma detection by NNs was conducted in [3].

For the segmentation task, authors in [9] implemented an efficient Adaptive Dual Attention Module, integrated into a dual encoder structure for automated SL segmentation with the best F1 score of 91.63%.

In the classification of SL by automatic learning methods, the extraction of features by NNs can be very useful. In this sense, in [10] the feature extraction by 17 pre-trained CNNs as feature extractors and the classification by 24 ML classifiers are analyzed. The best results were obtained by DenseNet201, as a feature extractor, and Fine KNN as a classifier (an accuracy of 92.34% on the ISIC 2019 dataset).

The potential of the deep learning (DL) concept to classify SLs was exploited in [11]. The classification system was obtained by training several state-of-the-art NNs. The accuracy obtained was 93% on the seven classes present in the ISIC archive, the dataset also used in our paper. An important factor that led to obtaining these classification performances was the use of non-visual data, related to the taxonomy of the disease as part of the decision-making ensemble. The final prediction was obtained by combining the individual results of all NNs, using the average values of the predictions for the best-performing four networks.

The authors in [12] used convolutional neural networks (CNNs) for melanoma recognition. A new regularizer which is based on the standard deviation of the weight matrix of the classifier is proposed to control the complexity of the classifier more precisely. A penalty is placed on the dispersion of the classifier weight values. Thus, the weights had relatively close values, and the correlation between these values was considered. The regulator is incorporated in each of the convolution layers to control the values of the kernel matrix.

In [13], an intelligent system based on deep learning techniques was proposed for the detection and differentiation of melanoma from benign lesions. By using a single innovative deep convolutional network, based on an encoding–decoding principle, important features of melanoma were extracted in a robust way. The encoder is responsible for learning general features (which include hairs) and location information. The decoder learns the contour characteristics of melanoma. After feature extraction, a pixel-level classifier was responsible for dividing the lesions into two categories. By comparison with the best-performing models from the ISIC Challenge 2017, it could be observed that this model presents considerably better sensitivity values compared to the other models.

Convolutional neural networks with a reduced number of parameters (lightweight) have become increasingly used in image classification architectures. Similarly, it was sought that the networks used in this work contain as few parameters as possible to reduce the calculation time. An efficient and compact classification model based on the MobileNet and DenseNet networks was proposed in [14]. A more efficient classification principle was introduced to improve discrimination between melanoma characteristics and classification accuracy. Also, a compact U-Net model based on feature extraction was designed to successfully segment the lesion area. The images were divided into pairs: positive (with melanoma) and negative (without melanoma) images, training the network by differentiating the features within the paired images. The training of the network was performed with pairs of images with melanoma and without melanoma.

Relatively recently in [15], the experimental performances in the context of melanoma lesion identification of the EfficientNetB6 network were presented in comparison with other established networks. Using the ISIC 2020 dataset and involving the transfer learning method starting from a pre-trained model on ImageNet, the results obtained were a few percent better than the results obtained, under the same conditions, using VGG-16 and VGG-19.

According to [16], the methods of assembling several neural networks trained separately have become more and more popular due to the superior performances they show in practice. The idea of ensemble learning is to combine several predictors to obtain an optimal convex combination, instead of using a single predictor. The idea of assembling several neural networks to improve the classification performance is a concept often used in specialized works in recent years and represents an important point of study, due to the many ways of creating ensembles. For example, to classify melanoma-type lesions, in [17] a system was proposed with a component responsible for segmenting regions of interest from medical images and a classification component represented by a set of five neural network models—DenseNet169, Inception-V3, Res-Net50, Inception-ResNet-V2, and Xception. The new trends in melanoma detection using NNs are highlighted in [3]. Also, for the detection of melanoma, in [18] an ensemble of two neural networks based on horizontal voting is proposed with good statistical performance (better than individual networks).

Choosing the dataset for learning, validation, and testing represents an important step in testing a proposed NN detection model. ISIC (International Skin Imaging Collaboration) represents the most used dataset of images with skin lesions for learning neural networks in the automatic detection of skin cancer in the early stages. The authors of [19] recommend methods for selecting efficient data subsets from the existing tens of thousands, eliminating irregularities (e.g., duplicates). The emphasis was placed on two major groups: melanoma and non-melanoma, considering the seriousness of the presence of melanoma. If it is necessary, an SL image augmentation method of the dataset based on artificial intelligence is proposed in [20]. Thus, a StyleGAN (Generative Adversarial Network) was used to generate new images and another NN (Dense-Net201) was used for classification.

In a recent work [21], we implemented, tested, and compared five NNs for SL detection and classification: ResNet-101, DenseNet-121, GoogLeNet, VGG16, and Mo-bileNetV2. We also considered seven SL classes from the HAM10000 dataset and an augmentation process to balance the number of SL in classes. The F1 score varied from 79% to 87% depending on the SL class and NN.

## 3. Materials and Methods

### 3.1. Dataset Used

**Figure 1:**
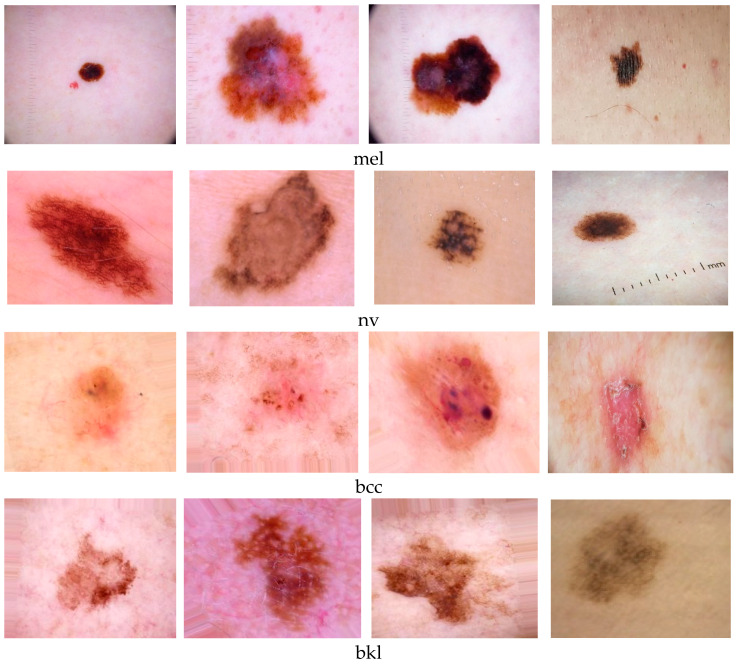
Different shapes and sizes of mel, nv, bcc, and bkl [23].

For this study, the dataset was composed of medical images collected from the HAM10000 [22] and ISIC Challenge 2019 [23] archives. Due to the imbalance between the number of images with the nv-type lesions and other types of lesions, approximately 20% of the nv images were randomly discarded. For the classes mel, bcc, and bkl, the dataset augmentation was achieved using [24]. Some examples with images from the four classes used are presented in Figure 1.

The first step in processing the dataset was the removal of duplicate images. There are overlaps between the two sets of data provided by the ISIC. In the metadata, each lesion is identified with a unique ID, as well as each image is identified with a unique ID. It was noticed that there are several images of the same lesion. To avoid possible errors, all the lesions that presented several images were removed.

The selected images were divided into 3 categories for the implementation of model training and image recognition:

- Training dataset—75% of total images;
- Validation dataset—15% of the total images;
- Test dataset—10% of total images.

As a rule, the ratio between learning, validation, and testing is 70/20/10. We chose 75/15/10 to increase the learning power.

For the classification of SLs from the four mentioned classes (mel, nv, bcc, and bkl), the images from the other three classes (akiec, df, and vasc) existing in the original dataset were completely abandoned. The image augmentation method was used for training so that the training data (after augmentation) consisted of 5047 images from the mel class, 5186 nv, 4815 bkl, and 4980 bcc. One of the reasons why these four classes were kept in a special way is the larger number of images present in the dataset, compared to images with the other types of lesions. The test dataset consisted of 342 images with bkl, 867 mel, 919 nv, and 485 bcc class lesions.

For the classification of SLs from the four mentioned classes (mel, nv, bcc, and bkl), the images from the other three classes (akiec, df, and vasc) existing in the original dataset were completely abandoned. The image augmentation method was used for training so that the training data (after augmentation) consisted of 5047 images from the mel class, 5186 nv, 4815 bkl, and 4980 bcc. One of the reasons why these four classes were kept in a special way is the larger number of images present in the dataset, compared to the images with the other types of lesions. The test dataset consisted of 342 images with bkl, 867 mel, 919 nv, and 485 bcc class lesions.

### 3.2. Neural Networks Used

To reduce the training time and to improve the performance of the models, the transfer learning method was used. Thus, the networks were trained on the original ImageNet dataset [24]. In the transfer learning process, the network starts from an initial point, with the weights of the neurons having values already tuned after training on another dataset (ImageNet). Furthermore, during the training for the problem in question, the optimization process of the network parameters will be carried out for a few layers smaller than the total number, with certain layers remaining constant.

The learning rate was initially set to 0.01 and did not remain fixed during training. To reduce the risk of overtraining the model on the training set, the learning rate was scheduled to be halved if, for two consecutive epochs, the validation metrics were not improved.

The validation metrics are the loss function value and the accuracy value. The chosen loss function is categorical_crossentropy. No performance differences were observed between using binary_crossentropy and categorical_crossentropy. Empirically, however, it was found that, for this problem, the entropy-based (probabilistic) loss functions are better than the regressive ones. The best model found from one epoch to another was retained and replaced only when a model with better validation metrics was found.

In this paper, two models of assembly of individual neural networks were studied for the purpose of decision fusion based on weights. To create the ensembles, three high-performance neural networks in object classification, with moderate complexity, were used: MobileNet [6], DenseNet 169 [7], and EfficientNet B2 [25]. The default size of the images used as input was 224 × 224 × 3 (where 3 is the number of color channels).

#### 3.2.1. MobileNetV2

**Figure 2:**
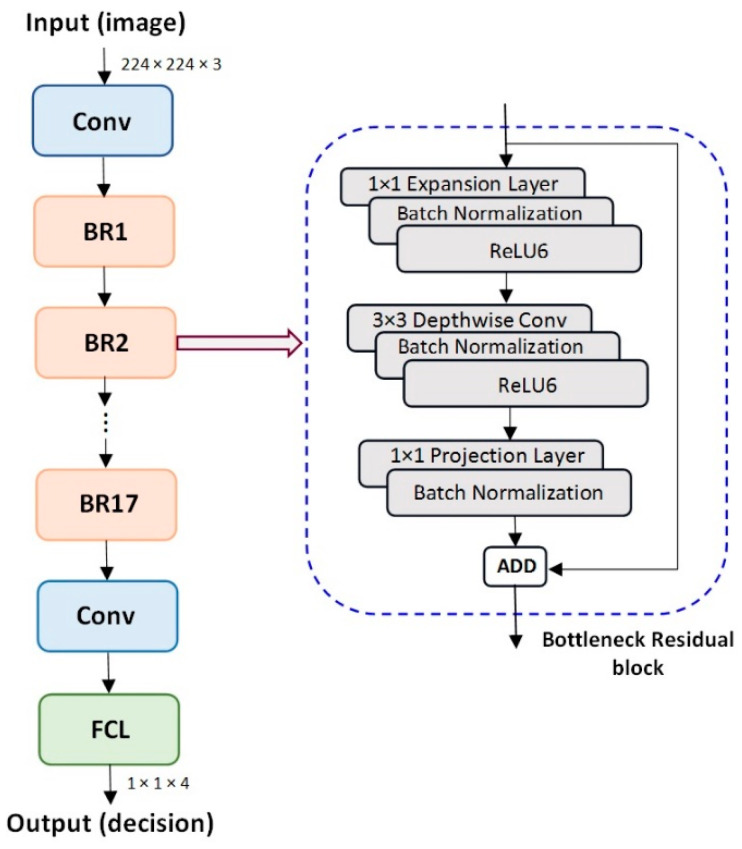
MobileNetV2 architecture (**left**) and Bottleneck Residual Block structure (**right**). Conv—convolutional layers, BR—Bottleneck Residual Block, FCL—Fully Connected Layer, ReLu—Rectified Linear Unit.

MobileNet aimed to reduce the complexity and the amount of memory involved. The important repetitive block (bottleneck layers) of MobileNet is the depthwise separable convolution that contains two modules: a depthwise convolution (a 3 × 3 spatial convolution) followed by a pointwise convolution (a 1 × 1 convolution to change the dimension) (Figure 2). This convolution, of 1 × 1 type, is applied for each color channel [6]. Then, a normal convolution combines all 3 channels and flattens them. Afterward, the pointwise convolution combines the outputs of the depth convolution. The characteristic of MobileNetV2 is that a residual block is formed as a bottleneck layer that contains, in addition to the two modules, another characteristic module at the input called the expansion layer.

The model is made up of parameters that vary between 1.7 and 6.9 million. The complete architecture of MobileNet is made of the sequences of layers presented in Figure 2, with the most important being the Bottleneck Residual Block (BR).

#### 3.2.2. DenseNet 169

**Figure 3:**
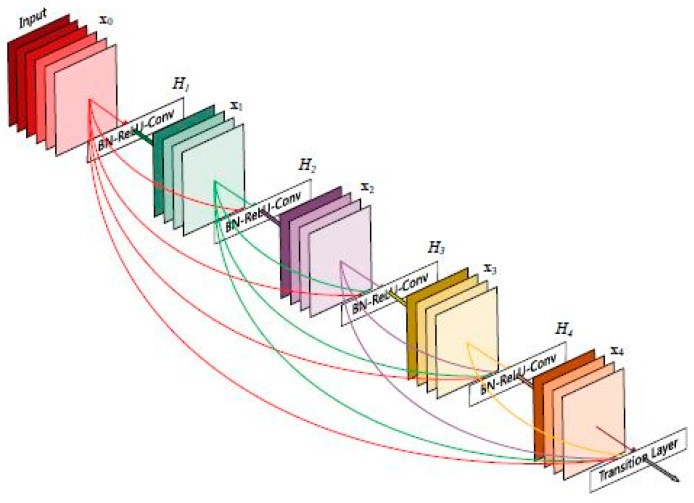
Five-layer DenseNet architecture [3].

The characteristic of DenseNet [7] is that each layer obtains information from all previous layers by concatenation and provides all feature maps (information) to the following layers (Figure 3). Thus, the input image passes successively through several layers of convolution, obtaining the feature maps.

The difference between the various implementations of the network (for example, DenseNet121 and DenseNet169) consists in the size of the model and implicitly in the accuracy.

#### 3.2.3. EfficientNet B2

**Figure 4:**
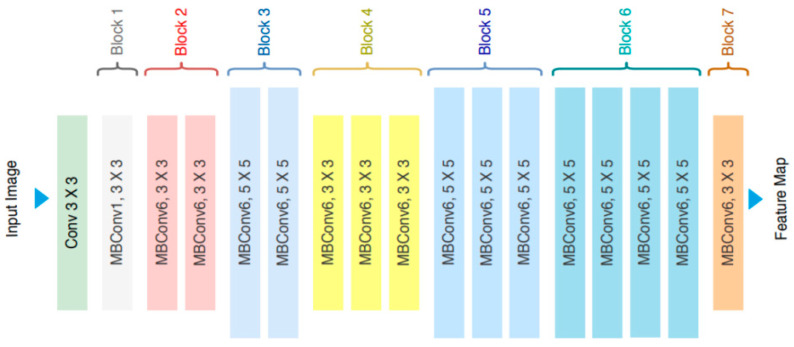
EfficientNet architecture [3].

EfficientNet represents a family of 8 convolutional neural networks obtained by scaling. For example, in [25], it is presented for scaling using a compound coefficient characterized by balancing the depth, width, and resolution of the neural network achieving a uniform scaling for all three dimensions. The most simple method of scaling a convolutional network to improve accuracy (by using more resources) is depth or width scaling. In Figure 4, the scaling types of a neural network are visually illustrated, along with the scaling by combining all the three types.

### 3.3. Methods of Combining Binary Models of the Same Network

We have analyzed three methods of combining the networks into an ensemble. The first ensemble was created from the binary models of the same network trained for pairs of lesions. The second ensemble was one created from three individual models of different networks for 4 classes of lesions, the decision being made by a score with multiple weights. The third model is the most complex and combines the two previous models, the global assembly being made up of 4 branches (one for each class) of individual binary ensembles of each network. The last two ensembles are analyzed in Section 4.

**Figure 5:**
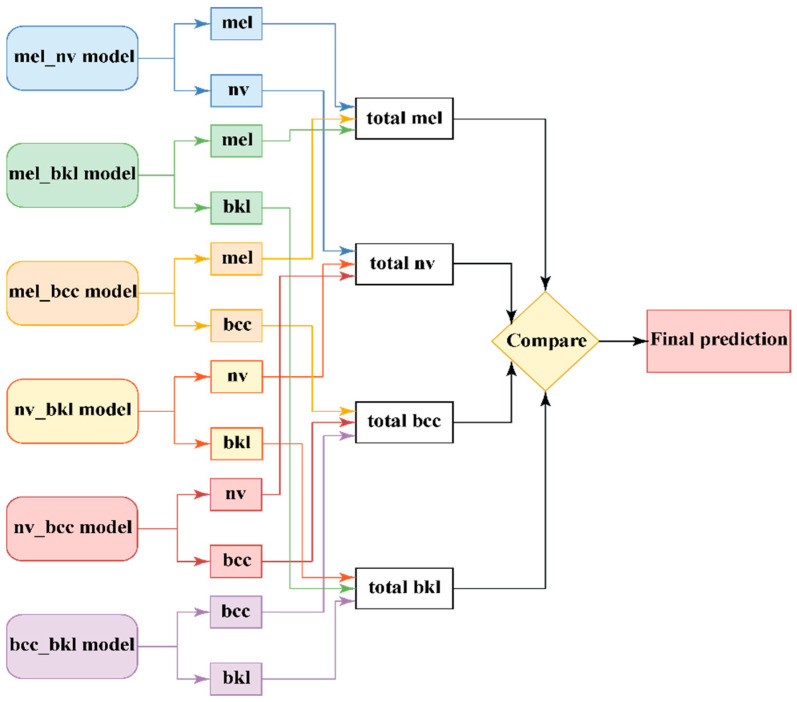
Prediction based on several different models of one NN.

To improve the accuracy of an initial model (NN), a classification strategy based on the training of several different models, specialized in the classification of only two classes (binary models), was tried. In total, four classes were identified; six such models that were able to classify only two classes (taken as combinations of all four classes) were trained for each CNN (Figure 5). Thus, for the four classes (mel, nv, bcc, and bkl), six models were created, called mel_nv, mel_bcc, mel_bkl, nv_bkl, nv_bcc, and bcc_bkl. The attached weights $w_{j,k}^{i}$ are noted and presented in Table 1. The weights are established empirically (in the validation phase) considering different performance indicators, such as accuracy, *F*1 score, etc.

Considering the decision $d(NN_{j}^{i})$ of the model *i* for the class *j* and the corresponding weights, a SCORE for each considered class, mel, nv, bcc, and bkl, are calculated in (1)–(4):

(1) $$\left(total\;mel\right)i=d\left(NN_{1}^{i}\right)\times w_{1,1}^{i}+d\left(NN_{2}^{i}\right)\times w_{2,1}^{i}+d\left(NN_{3}^{i}\right)\times w_{3,1}^{i}$$

(2) $$\left(total\;nv\right)i=d\left(NN_{1}^{i}\right)\times w_{1,2}^{i}+d\left(NN_{4}^{i}\right)\times w_{4,1}^{i}+d\left(NN_{5}^{i}\right)\times w_{5,1}^{i}$$

(3) $$\{total\;bcc)i=d\left(NN_{3}^{i}\right)\times w_{3,2}^{i}+d\left(NN_{5}^{i}\right)\times w_{5,2}^{i}+d\left(NN_{6}^{i}\right)\times w_{6,1}^{i}$$

(4) $$\left(total\;bkl\right)i=d\left(NN_{2}^{i}\right)\times w_{2,2}^{i}+d\left(NN_{4}^{i}\right)\times w_{4,2}^{i}+d\left(NN_{6}^{i}\right)\times w_{6,2}^{i}$$

The decision is taken by the maximum of the previous SCORE values.

After voting among the six models and thanks to the high chances of correct prediction from the models capable of recognizing the type of lesion from the test image, the final prediction will be as accurate as possible.

### 3.4. Performance Metrics

Performance metrics have an essential role in the analysis of the obtained results and the appreciation of neural networks. Depending on the quality of the performance metrics used, the quality of the results obtained can also be determined. The most frequently used performance metrics are presented in Table 2; the meaning of the notations being the following: TP—True Positive, TN—True Negative, FN—False Negative, and FP—False Positive. The performance metrics can be used to establish the weight of each NN.

In the case of the multiclass experiment for the specific weights, we used the multiclass F1 score as (5)

(5) $$F1j=\frac{2TPj}{2TPj+\sum^{\;}FP+\sum^{\;}FN}$$

where *TPj* is the true positive for the *j* class, $\sum^{\;}FP$ is the sum of false positive cases, and $\sum^{\;}FN$ is the sum of false negative cases for the *j* class.

### 3.5. Software and Hardware Used

The software tools and hardware resources used to implement the assembly of classification of lesions from medical images were the Python 3.6 programming language, the Jupyter Notebook development environment, and the Keras machine learning framework [26]. It ran on the well-known TensorFlow machine learning platform [27].

To put into context the training times that will be presented in the results section, the machine on which the model was trained will be presented: a laptop with i7 processor generation 10, 16 GB RAM memory, and a dedicated video card with 4 GB Nvidia GTX 1650Ti memory.

## 4. Ensemble Implementation

To increase the detection system performance, we proposed two multi-NN ensemble implementations. The first (Ensemble Model 1) is based on the direct decisions of each NN, multiplied by the associated weights. The second (Ensemble Model 2) considers binary models for each NN and the binary results are collected from all NNs.

### 4.1. Ensemble of Simple Models with Multiple Weights (Ensemble Model 1)

For the implementation of Ensemble Model 1, the three mentioned networks were chosen (MobileNetV2, DenseNet169, and EfficientNet B2), which were trained independently using the same training and validation dataset. Initially, the models were pre-trained on the ImageNet dataset, and then, the procedure called transfer learning was applied, which led to reduced training times and improved accuracy. To obtain better performance, all models were trained for a slightly longer time. Thus, the number of training epochs chosen was 25 to maximize the chance of finding a better configuration.

The final model was created by assembling the three models obtained from the individual training. This was performed to eliminate prediction errors through the voting effect.

Before training, however, several computationally expensive layers were removed from those initially present in each network. The reason was to reduce training time. From the examination of the results, it was found that the partial elimination of the layers leads to a better ratio between the training time and the results obtained.

After the training of the three models, their ensemble was carried out to improve the total accuracy and the *F*1 score for each class. The independent predictions of the networks were used and multiplied by corresponding weights. The weight values of predictions are considered the *F*1 scores. More precisely, the model with the higher *F*1 score will have a greater weight than the other models. The result of combining the three predictions proved to be better than any of the predictions taken independently. We chose the *F*1 score to have multiple weights for one NN, depending on the predicted class.

**Figure 6:**
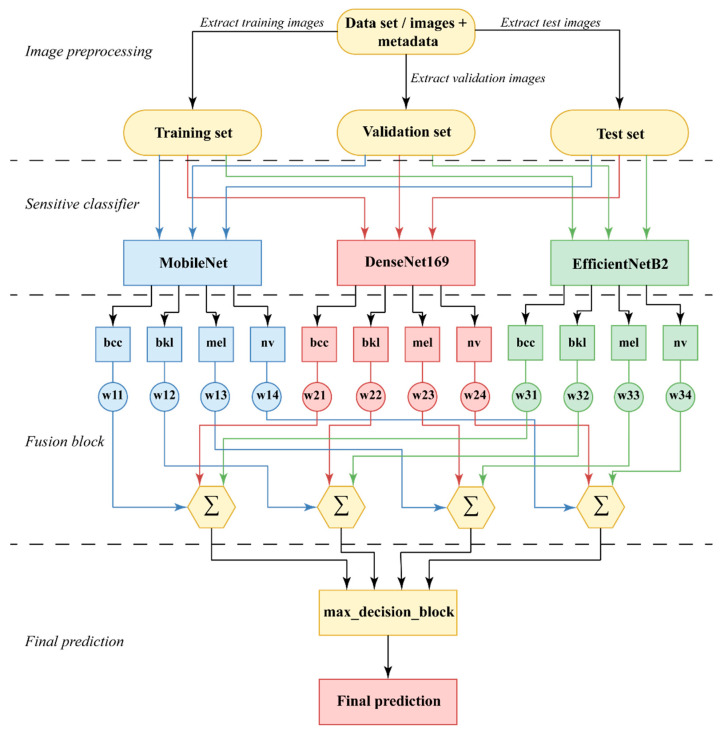
Diagram of application weights and classification of images, Ensemble Model 1.

The most important characteristic of this group is the fact that the emphasis was placed on the weighting of the votes, according to the individual performances of each model compared to the performances of the other models. This rationale should lead to better results by assigning proportional decision-making powers. The process of building the final classification model thus presented is illustrated in Figure 6.

The system has four sections: (a) image preprocessing, in which data is also separated for the three stages of operation (training, validation, and testing); (b) sensitive classifier, having three networks with a classifier function; (c) fusion block, to obtain decisions on each branch and summing up the partial predictions for each class; and (d) final prediction, for selecting the maximum value among the four partial predictions/class. For this task, the matrix of partial predictions P (6) corresponding to each NN, and each class (12 elements) is calculated. This matrix will be multiplied member by member with the weight matrix (7). The weight matrix is also 3 × 4 in size. The elements of the weight matrix W (w_i,j_) are calculated for each network *i* (*i* = 1, 2, 3) and each class *j* (*j* = 1, 2, 3, 4) and correspond to the *F*1 score *F1*ij, as in (1). The network correspondence is 1—MobileNet, 2—DenseNet169, and 3—EfficientNetB2. The class correspondence is 1—bcc, 2—bkl, 3—mel, and 4—nv.

Prediction matrix:

(6) $$P=\left(\begin{array}{cccc} NN1\_bcc & NN1\_bkl & NN1\_mel & NN1\_nv\\ NN2\_bcc & NN2\_bkl & NN2\_mel & NN2\_nv\\ NN3\_bcc & NN3\_bkl & NN3\_mel & NN3\_nv \end{array}\right)$$

where MobileNet = *NN*1 (network 1), DenseNet = *NN*2 (network 2), and EfficientNet = *NN*3 (network 3).

This matrix will be multiplied member by member with the weight matrix. The weight matrix is also 3 × 4 in size (2).

Weight matrix is defined as

(7) $$W=\left(\begin{array}{cccc} w11 & w12 & w13 & w14\\ w21 & w22 & w23 & w24\\ w31 & w32 & w33 & w34 \end{array}\right)$$

The result—prediction of the lesion is the class with the maximum value of one of the expressions (4)*–*(7).

(8) $$bcc_{pred}=NN1\_bcc\times w11+NN2\_bcc\times w21+NN3\_bcc\times w31$$

(9) $$bkl_{pred}=NN1\_bkl\times w12+NN2\_bkl\times w22+NN3\_bkl\times w32$$

(10) $$mel_{pred}=NN1\_mel\times w13+NN2\_mel\times w23+NN3\_mel\times w33$$

(11) $$nv_{pred}=NN1\_nv\times w14+NN2\_nv\times w24+NN3\_nv\times w34$$

The final prediction corresponds to a maximum value of (8)–(11).

### 4.2. Ensemble of Binary Models with Multiple Weights (Ensemble Model 2)

**Figure 7:**
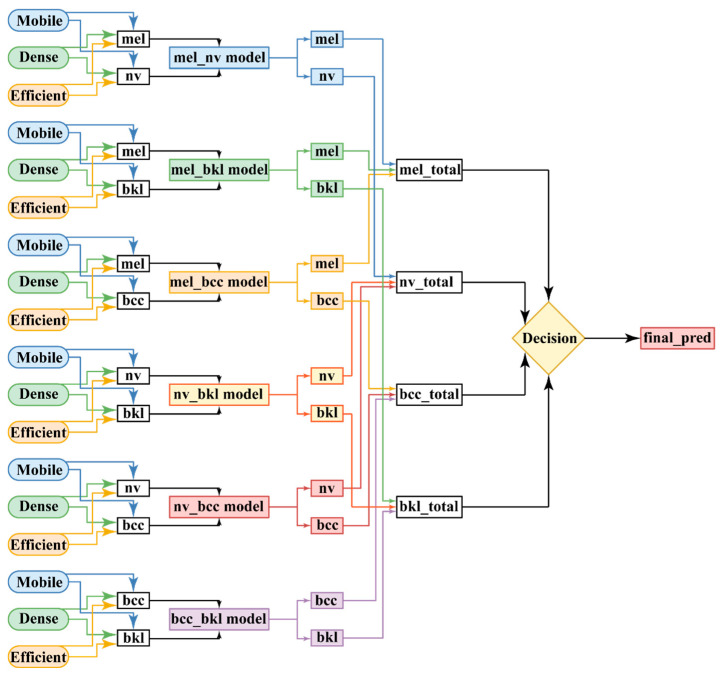
Diagram of the ensemble with binary models and multiple weights (Ensemble Model 2).

This method is the most complex and resource-consuming and leads to obtaining the best accuracy values for identifying the four classes of lesions. The method is obtained by combining the concepts used to create the previous two ensembles (Figure 5 and Figure 6). More precisely, the improvement consists of replacing each of the three individual models from Figure 6 with a binary ensemble of the form in Figure 5. The result is an ensemble with 18 individual binary classifiers, as shown in Figure 7. We found that the individual improvement, even with small percentages of the models constituting the ensemble from Figure 6, leads to a satisfactory improvement in the final accuracy. We decided that a method by which the F1 score of the six constituents of the ensemble would be the use of the final ensemble by voting, through the mechanism with multiple weights.

Thus, for the Ensemble Model 2, the total score of weighted votes from the three NNs for each class is calculated: total mel, total bkl, total bcc, and total nv. Taking advantage of the high accuracy values of each individual model (binary classification), the total accuracy will be increased. The classification of a lesion image in the test stage is performed by considering the partial results of each of the six models, three of them being definitely trained to recognize it.

In the section responsible for assembling the models, the values of the predictions multiplied by the corresponding weights for each of the four classes obtained from each model are added. The results of the meetings are compared, and the final decision is made. This is to mimic the behavior of a random forest classifier. It represents a forest of decision trees that will make a prediction based on the vote. Their cumulative prediction will be more correct than the prediction of everyone. This assembly is built to cover the problems of each individual model by making a group decision. In general, the richer the ensemble would have been, the better the accuracy would have been, but this would have led to an increase in training time.

The binary models of the three networks are grouped into branches corresponding to the considered pairs: mel_nv, mel_bkl, mel_bcc, nv_bkl, nv_bcc, and bcc_bkl. Then, the tree is reduced to four branches, mel_total, nv_total, bcc_total, and bkl_total, from which the decision is taken by maximum selection. They are evaluated by the sum of the corresponding binary models (12)–(15). The decision is taken by the maximum from the previous values.

(12) mel total=mel nv model+mel bkl model+mel bcc model

(13) nv total=mel nv model+nv bkl model+nv bcc model

(14) bcc total=mel bcc model+nv bcc model+bcc bkl model

(15) bkl total=mel bkl model+nv bkl model+bcc bkl model

Adapting the voting method with multiple weights in the case of binary models, the total SCORE for each class is computed as in (16)–(19).

(16) $$mel\_total=\overset{3}{\underset{i=1}{\sum }}d\left(NN_{1}^{i}\right)\times w_{1,1}^{i}+\overset{3}{\underset{i=1}{\sum }}d\left(NN_{2}^{i}\right)\times w_{2,1}^{i}+\overset{3}{\underset{i=1}{\sum }}d\left(NN_{3}^{i}\right)\times w_{3,1}^{i}$$

(17) $$nv\_total=\overset{3}{\underset{i=1}{\sum }}d\left(NN_{1}^{i}\right)\times w_{1,2}^{i}+\overset{3}{\underset{i=1}{\sum }}d\left(NN_{4}^{i}\right)\times w_{4,1}^{i}+\overset{3}{\underset{i=1}{\sum }}d\left(NN_{5}^{i}\right)\times w_{5,1}^{i}$$

(18) $$bcc\_total=\overset{3}{\underset{i=1}{\sum }}d\left(NN_{3}^{i}\right)\times w_{3,2}^{i}+\overset{3}{\underset{i=1}{\sum }}d\left(NN_{5}^{i}\right)\times w_{5,2}^{i}+\overset{3}{\underset{i=1}{\sum }}d\left(NN_{6}^{i}\right)\times w_{6,1}^{i}$$

(19) $$bkl\_total=\overset{3}{\underset{i=1}{\sum }}d\left(NN_{2}^{i}\right)\times w_{2,2}^{i}+\overset{3}{\underset{i=1}{\sum }}d\left(NN_{4}^{i}\right)\times w_{4,2}^{i}+\overset{3}{\underset{i=1}{\sum }}d\left(NN_{6}^{i}\right)\times w_{6,2}^{i}$$

The significance of notations in the previous equations is the following:

- $NN^{1}$ is MobileNetV2, $NN^{2}$ is DenseNet169, and $NN^{3}$ is EfficientNetB2;
- $w_{j,k}^{i}$ are similar notations as in Equations (1)–(4), having the *i* index customized for the respective network.

## 5. Experimental Results

### 5.1. Results of Simple Models with Multiple Weights (Ensemble Model 1)

**Figure 8:**
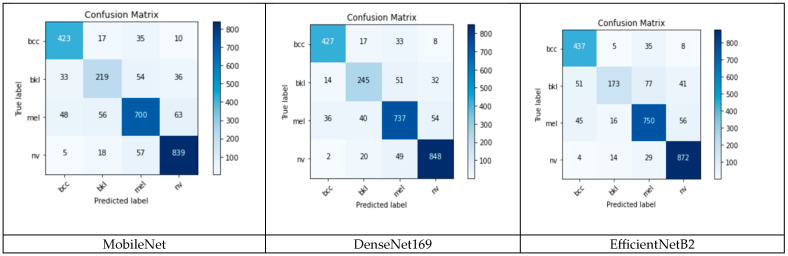
The confusion matrices for the models used in the ensemble.

To establish the weights of each individual network and each class, confusion matrices are evaluated in the validation phase (Figure 8).

The F1 score for each model and each class was calculated from (1), considering the confusion matrices from Figure 8. It can be observed that the *F*1 score is different for individual NN and class (Table 3). These *F*1 scores were considered as weights associated with an individual NN and class.

Mean accuracy for the individual classifiers (networks) and ensemble is calculated in the validation phase and is shown in Table 4. It can be observed that the ensemble accuracy is better than individual accuracy.

### 5.2. Results of Ensemble of Binary Models with Multiple Weights (Ensemble Model 2)

**Figure 9:**
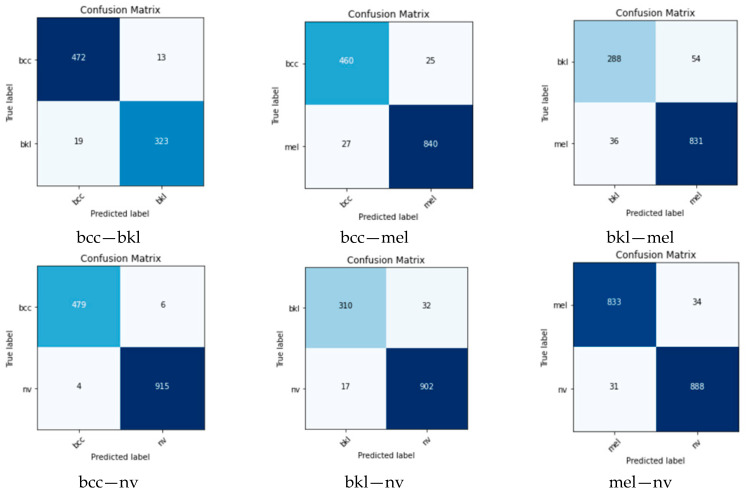
Confusion matrices for different binary branches.

Considering separately the binary ensembles of the three NN, for the four classes mentioned in the previous chapter, it can be observed that the results are very good for the six branches of the final ensemble and better than each individual ensemble (Table 5). The accuracy of the global branches was calculated according to the confusion matrices in Figure 9. Large differences between the branches are also observed. For example, on the bek—mel branch, the lowest accuracy is obtained (92.56%) and on the bcc—nv branch, the highest accuracy is obtained (98.28%).

**Figure 10:**
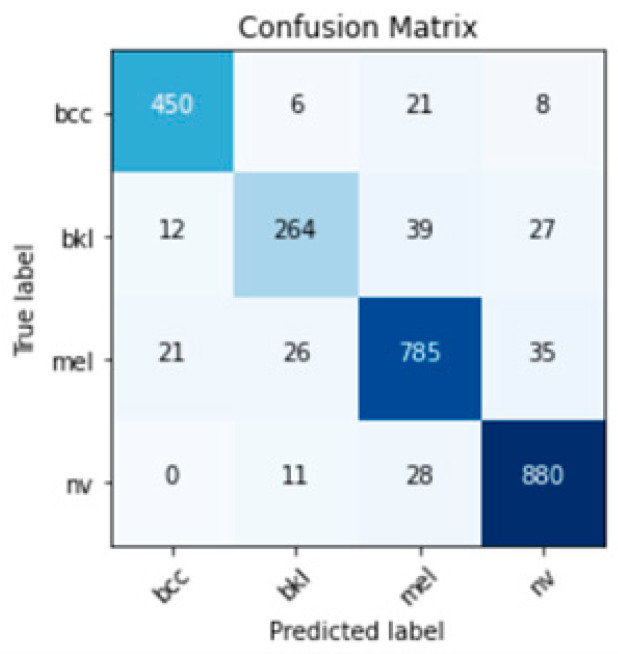
Confusion matrix for Ensemble Model 2.

The final ensemble for classifying the skin lesion in one of the four classes has a weaker result than the binary branches, which was expected because the number of classes increased from two to four. Thus, from the global confusion matrix (Figure 10), F1 scores were evaluated for the four classes (Table 6), with an average accuracy of 91.04%. Table 6 compares the two performances for Ensemble Model 1 and Ensemble Model 2. For all classes, the statistical indicators of the model with subsets of binary models are better than those of Ensemble Model 1. Table 6 shows the number of images from the dataset in each class for the testing phase. The results per class differ quite a lot (F1 score), with bkl being the weakest and for nv the best.

## 6. Discussion

The results obtained highlighted the fact that the use of binary models improved the performance of both individual and global classifiers as shown in Table 5. A deficiency of the dataset used was imbalances between classes (quite a different number of class representatives). This will be remedied in future studies by choosing a dataset with a sufficiently large and equal number of images in each class. This may be another explanation for the fact that for bkl the worst performances were obtained.

In Table 7, a comparison is presented between the proposed work and some important works from the current literature in the field. The table was created based on the information presented in the mentioned works and with the help of the information from [3].

It can be seen from Table 7 that the results obtained when identifying melanoma from medical images are good, falling within the level obtained in [18] because the last one is a binary classifier (melanoma and non-melanoma). The same can be said for the classification of skin lesions in several classes. A system based on decision fusion was also proposed in this work, so a comparison between the two is at hand. The superior preprocessing of the images along with the variety of identification methods in the final system represents the strengths of this work. On the other hand, the larger number of training data and different ensembles proposed lead to comparable results.

Good results were also obtained in [29] by a system also based on the fusion; this time the features were obtained both manually and automatically. Me and nv-type lesions were classified, and the result was somewhat lower than the one obtained in the first case study in the proposed paper.

Regarding the classification of several types of lesions, in [8], a somewhat better result is obtained than the result presented in this proposed paper. Due to the use of a variation in the evolutionary PSO optimization algorithm, the parameters of the deep neural network, as well as the number of layers, could be optimized with increased flexibility. On the other hand, in [8], the aim was to obtain a good value of accuracy but with the balancing of sensitivities for each class to be identified. The final result is somewhat weaker than the result obtained in the proposed work, but, as can be seen from Table 6, the sensitivity for the bkl class is somewhat weaker than for the case of the other classes of lesions.

Therefore, all the works listed in the table above present systems capable of identifying and classifying skin lesions with very good accuracy. Each work has certain advantages and disadvantages that lead to close results following different approaches. Among the methods that could lead to improvements in the results of the proposed work are the development of a more laborious preprocessing stage and the development of a cost function that considers the particularities of the problem.

## 7. Conclusions

In this work, two classification methods based on ensembles of convolutional neural networks and decision fusion were analyzed. The lesions with the best representation in terms of the training data were chosen. Two ways of assembly by voting were exploited. The best result was obtained by a global ensemble composed of a number of networks configured on binary classifications (individual branches); the training of which took approximately 30 h. The relatively high calculation time was because a high-performance computer network was not available. Each binary model of the networks (18 in total) was learned separately so that the total time was obtained by concatenating the 18 learning times. This mode does not affect the overall accuracy of the system, because the images are not dynamic, but static. The accuracy of 91.04% is promising, considering the possibilities of improving the preprocessing stage by eliminating images that present noise. The aim of the work was to explore the voting assembly methods of neural networks. The results obtained by the individual training of some networks for the classification of four types of lesions were considerably lower than the results obtained by each created ensemble. While the training of a network is limited by over-adaptation, the ensembles show superior robustness through the ability to eliminate the defects of the networks involved by the other members.

## Figures and Tables

**Table 1:** The attached weights $w_{j,k}^{i}$ for *NN^i^*.

Model $NN_{}^{i}$	Weight mel	Weight nv	Weight bcc	Weight bkl
Model mel_nv $NN_{1}^{i}$	$w_{1,1}^{i}$	$w_{1,2}^{i}$	_	_
Model mel_nv $NN_{2}^{i}$	$w_{2,1}^{i}$	_	_	$w_{2,2}^{i}$
Model mel_nv $NN_{3}^{i}$	$w_{3,1}^{i}$	_	$w_{3,2}^{i}$	_
Model mel_nv $NN_{4}^{i}$	_	$w_{4,1}^{i}$	_	$w_{4,2}^{i}$
Model mel_nv $NN_{5}^{i}$	_	$w_{5,1}^{i}$	$w_{5,2}^{i}$	_
Model mel_nv $NN_{6}^{i}$	_	_	$w_{6,1}^{i}$	$w_{6,2}^{i}$

**Table 2:** Statistical performance indicators for evaluating models (binary case).

Name	Formula	Name	Formula
Sensibility	$TPR=\frac{TP}{TP+FN}$	Specificity	$TNR=\frac{TN}{TN+FP}$
Precision	$PPV=\frac{TP}{TP+FP}$	Accuracy	$ACC=\frac{TP+TN}{TP+TN+FP+FN}$
F1 score	$F1=\frac{2TP}{2TP+FP+FN}$	Jaccard index	$JI=\frac{TP}{TP+FP+FN}$

**Table 3:** *F*1 score (as associated weights) evaluated from the confusion matrices.

Network	Class			
	mel	nv	bcc	bkl
MobileNet	w_11_ = 0.82	w_12_ = 0.90	w_13_ = 0.85	w_14_ = 0.67
DenseNet 169	w_21_ = 0.85	w_22_ = 0.91	w_23_ = 0.89	w_24_ = 0.74
EfficientNetB2	w_31_ = 0.85	w_32_ = 0.92	w_33_ = 0.86	w_34_ = 0.63

**Table 4:** Mean accuracy for individual classifiers and Ensemble model 1.

Model	MobileNetV2	DenseNet 169	EfficientNetB2	Ensemble
Accuracy	83.46%	85.49%	86.37%	89.62%

**Table 5:** Accuracy of individual binary classifiers.

Binary Classifiers	MobileNet	DenseNet169	EfficientNetB2	Globalbranch
bcc—bkl	92.26%	92.86%	94.31%	96.48%
bcc—mel	93.93%	94.89%	94.30%	96.15%
bkl—mel	88.91%	89.00%	91.15%	92.56%
bcc—nv	98.15%	98.50%	99.07%	99.28%
bkl—nv	95.48%	95.79%	95.24%	96.11%
mel—nv	94.45%	95.40%	94.17%	96.36%

**Table 6:** Comparison between the test performances of multi-network ensemble models.

Class	Ensemble Model 1		Ensemble Model 2		Support (Images)
	F1 Score	Accuracy	F1 Score	Accuracy	
mel	84.56%	86.80%	90.23%	91.04%	867
nv	92.60%		94.17%		919
bkl	72.87%		81.36%		342
bcc	86.30%		92.97%		485
					Total = 2613

**Table 7:** Synthesis for the purpose of comparison with several important works in the field.

Ref.	Goal	Description	NN	Data Base	ACC
[28]	Classification of skin lesions with minimization of the sensitivity variant	Hybrid data balancing system between classes	EfficientNet-B4	ISIC Challenge Dataset	89.97%
[8]	Variation in the PSO optimization algorithm for the segmentation and classification of skin lesions	Combination of HLPSO, convolutional neural network and K-Means algorithm	DCNN	ISIC 2017	91.37%
[29]	System based on the fusion of information obtained manually and from different neural networks	Combining the ABCD algorithm with the features extracted by DCNN networks	VGG-16, VGG-19, MobileNet, ResNet-50, Xception, Inception v3, DenseNet-201	HAM10000	92.40%
[30]	CNN model using a triplet cost function for classification of facial skin lesions	Fine tuning of ResNet152 and Inception ResNet-v2 layers	ResNet152, Inception ResNet-v2	Hospital from Wuhan, China	87.42%
[31]	The integration of different neural networks in a decision system with weights depending on individual performances	Global classifier implemented with the help of individual classifiers.	CNN, GoogleNet, ResNet101, NasNet-Large, Perceptron	PH2, ISIC 2019	88.33–93.33%
[18]	Detection of two classes of SL: melanoma and non-melanoma by an ensemble of two NNs	Using of Horizontal Voting of two NNs	MobileNet, DenseNet169	HAM10000, ISIC 2019	96.06%
Our	Classification of several skin lesions including melanoma	Exploring two voting assembly methods	EfficientNet DenseNet MobileNet	HAM10000 and ISIC	91.04%

## Data Availability

The data presented in this study are available in this article.
